# ‘Instruments are good at eliciting information; scores are very dangerous’: The perspectives of clinical professionals regarding neurodevelopmental assessment

**DOI:** 10.1177/13623613221121413

**Published:** 2022-09-25

**Authors:** Barry Coughlan, Matt Woolgar, Emma JL Weisblatt, Robbie Duschinsky

**Affiliations:** 1 University of Cambridge, UK; 2 King’s College London, UK; 3 Cambridgeshire and Peterborough NHS Foundation Trust, UK

**Keywords:** attention deficit hyperactivity disorder, autism, clinical reasoning, qualitative, decision-making, diagnostic upgrading

## Abstract

Clinical judgement is a crucial part of autism and attention deficit hyperactivity disorder (ADHD) assessments. This study aims to harness insights from psychology and sociology to explore clinical perspectives and assessment practices of autism and attention deficit hyperactivity disorder. Qualitative interviews were conducted with health care professionals (n = 17). Interviews were conducted between January and May 2019. Data were analysed using a thematic approach. Themes were sorted into two interrelated categories (1) approaches to diagnosis (2) elements of diagnosis. Approaches to diagnosis were comprised of the following subthemes: nature of diagnosis, application of diagnosis: natural and pragmatic, revisiting conceptualisations: sub-optimal practice & unhelpful diagnoses, autism and attention deficit hyperactivity disorder. Elements of diagnosis contained three related subthemes: contextualising standardised assessments, triangulating material, organisational factors. There is sometimes a pragmatic as well as nosological dimension to diagnosis. Competing desires for consistency and utility add further complexity to neurodevelopmental assessment.

**Lay abstract:**

Autism and attention deficit hyperactivity disorder are common behaviourally diagnosed conditions. One of the key aspects of diagnosis is clinical judgement. Yet despite decades of research, it is only in recent times that researchers have started exploring clinicians’ perspectives on diagnosing these conditions. We aimed to add to this body of knowledge by conducting interviews with 17 experienced health care professionals in the United Kingdom to hear their perspectives on diagnosing autism and attention deficit hyperactivity disorder. Clinicians reflected that for some children and young people, diagnosis is reasonably straightforward; however, in other situations, decisions are made on more pragmatic grounds (i.e. will this be helpful). We identified some differences of opinion between professionals and organisation which adds to the complexity of applying a diagnosis. We recommend several areas for future research and point to some practical and philosophical implications of the work.

Assessing autism and attention deficit hyperactivity disorder (ADHD) can be challenging. Clinical profiles are often heterogeneous, and comorbidities with other neurodevelopmental (e.g. intellectual disability) and psychiatric conditions (e.g. anxiety) are common (Lai et al., 2019; Luo et al., 2019; Simonoff et al., 2008; Thapar & Cooper, 2016). Adding to the complexity, an overwhelming body of evidence indicates that features of autism and features of ADHD frequently co-occur (Leitner, 2014; Ronald et al., 2014; Simonoff et al., 2008; Stevens et al., 2016; Taurines et al., 2012; Zablotsky et al., 2020).

Best practice guidelines (The National Institute for Health and Care Excellence, 2011, 2018) suggest that core features of a ‘robust’ assessment include multidisciplinary assessment, comprehensive physical examination, information from multiple respondents (e.g. individual, parent, teacher), observations of behaviour in different contexts (e.g. home, clinic, school), a detailing of the developmental history and standardised measures of symptomology (e.g. behavioural rating scales, observations, clinical interviews), and, ultimately, clinical judgement. Yet only recently have clinicians’ perspectives and experiences applying these diagnoses generated scholarly interest (Hayes et al., 2021; Turowetz & Maynard, 2016, 2019).

In his seminal work on social construction, Hacking (1999) offers a number of reflections that are potentially helpful when considering the ontological status of autism and ADHD. In particular, Hacking offers a potentially useful distinction between the object (i.e. the diagnosis) and the idea of the object (e.g. the classification of autism or ADHD). According to Hacking, objects (e.g. autism or ADHD) might well be ‘real’ in the common-sense meaning of the word (Hacking, 1999, p. 21), whereas ideas about objects (e.g. the classification of autism; a child with ADHD) are shaped by various factors including standardised assessment tools, best practice guidelines, media discourses and advocacy groups. Turowetz and Maynard (2016) discuss a narrative practice they call ‘category attribution’ which involves comparing descriptions of the child’s behaviour or development is with a broad range of reference groups (e.g. typically developing children or children with autism). The authors describe two forms of category attribution: (1) situating a child within a given category and (2) pointing to evidence of the ill-fit of a child of a category. Recently, Turowetz and Maynard (2019) applied conversation analysis to psychological reports and clinical discourses in the autism assessments. The authors suggest that reports tend to spotlight the child’s behaviour while playing down contextual factors. This work offers important insights into the narrative practices through which reasoning is expressed when health care professionals (HCPs) are evidencing their diagnosis. Yet, the issues of diagnostic uncertainty are not in view.

Most research on neurodevelopmental assessment has focused on developing standardised tools to quantify symptomology. For autism, the Autism Diagnostic Observation Schedule-2 (ADOS-2; Lord et al., 2012) and the Autism Diagnostic Interview–Revised (ADI-R; Lord et al., 1994) are often regarded as the ‘gold standard’ (Falkmer et al., 2013) psychometric assessments. Unlike autism, there is less consensus regarding the ‘gold standard’ psychometric assessment for ADHD symptomology. Nevertheless, an array of behavioural rating scales has been mapped to ADHD diagnostic criteria. These tools include the Strengths and Difficulties Questionnaire (SDQ; Goodman, 2001; Goodman, Ford, Simmons, et al., 2000), Connor’s rating scale (Conners, 2008) and development and well-being assessment (DAWBA; Goodman, Ford, Richards, et al., 2000).

Several studies have used self-report survey methods to explore HCP’s assessment practices regarding autism and ADHD. Rogers et al. (2016), for instance, surveyed UK-based professionals about their experiences diagnosing autism. Interestingly, this survey asked professionals about their experiences of ‘upgrading’ a diagnosis. This the authors define as the giving of a positive diagnosis ‘in the face of an unclear presentation or patients failing to meet criteria on diagnostic tools’ (Rogers et al., 2016, p. 829). Overall, 32% of participants reported that they would ‘never’ upgrade to a diagnosis of autism. By contrast, half indicated that they had done this infrequently, and 12% indicated this was a ‘frequent’ or ‘very frequent’ practice. Participants ‘upgraded’ for an array of reasons, including to help children gain access to supports and educational placements, conflicting views within the diagnostic team and shortcomings of standardised tools. Yet, the operational definition of ‘upgrading’ in this study requires attention. As discussed previously, an individual not meeting diagnostic thresholds on an assessment tool does not mean that they do not have autism (The National Institute for Health and Care Excellence, 2011). Therefore, as the authors acknowledge, these responses do not necessarily indicate that professionals are relaxing diagnostic thresholds. Instead, it might indicate conflicts between clinical judgement and the outcomes of standardised assessments.

Nevertheless, earlier work (Skellern et al., 2005) does point to times where diagnostic thresholds have been relaxed. In their article, the operational definition of upgrading included exaggeration of symptoms. Overall, 60 (58%) of HCPs indicated that, on at least one occasion, they had given a diagnosis of ‘on the autism spectrum’ so that the child could avail of educational support. Of these, four professionals indicated they had given a diagnosis in a context where they were confident that the child did not meet diagnostic criteria. Relatedly, a subsequent survey in Australia (Taylor et al., 2016) reported that nearly half (n = 97) of professionals had given a ‘provisional diagnosis of autism’ in diagnostically uncertain cases.

From a psychometric perspective, the issue of an individual not fulfilling diagnostic thresholds on standardised assessments speaks to practitioner concerns regarding test sensitivity. In contrast to ‘upgrading’ a diagnosis, therefore, the diagnosis might be ‘downgraded’. Downgrading, for instance, might happen when despite meeting criteria on an assessment tool, a diagnosis is not given due to concerns about, in psychometric parlance, test specificity.

## Aim

This study aims to explore clinical perspectives and assessment practices of autism and ADHD through qualitative interviews with experienced HCPs. We illuminate some considerations around assessment and challenges that HCPs might encounter.

## Method

### Overview

We conducted semi-structured interviews with 25 HCPs, including 8 general practitioners from the National Health Service (NHS) Trusts across the United Kingdom. Findings regarding referral pathways and differential conceptualisation of attachment difficulties are presented elsewhere. Therefore, this article will focus on the interviews with the 17 HCPs from child and adolescent mental health services (CAMHS) and the neurodevelopmental teams and their considerations around neurodevelopmental assessment. Therefore, the interviews with general practitioners are not included in the current analysis. Ten participants were psychologists; two were psychiatrists; two were paediatricians; three were cognate allied HCPs. Interviews ranged from 48 to 69 min. Five participants were male, and 12 were female. Interview topics included professional background, routine clinical work, case study and referral pathways. University Ethics, The Health Research Authority and local NHS research and development teams approved the study prior to data collection. Data were collected between January 2019 and May 2019. BC interviewed all participants, and interviews took place in person or remotely (e.g. by phone or Skype). All interviews were audio-recorded and transcribed verbatim by either BC or professional transcription service. All participants gave written informed consent prior to the interview, and verbal consent was confirmed at the end of each interview.

### Procedures

A flexible topic guide was prepared by the authors. This was based on current literature and clinical experience. Topics included professional background, routine clinical work, hypothetical case study (General Practitioner case study or CAMHS case study) and referral pathways. The guide was piloted with three HCPs. An information power approach was used to inform sampling size. This interview guide including the case study can be found in Supplement 1. All participants were asked not to reveal any personally identifiable information about any child or families they work with at the start of the interview, and questions about routine clinical work was prefaced with this reminder. Still, some changes have been made to some of the reflections (e.g. particular interests of a child) to ensure complete anonymity. To this end, at times, gender pronouns have been changed or removed.

### Setting and recruitment

As discussed above, HCPs were recruited from local CAMHS, neurodevelopmental and national specialist services. These are multidisciplinary teams, including psychologists, psychiatrists, nurses, speech and language therapists, occupational therapists, paediatricians and psychotherapists. In the United Kingdom, an assessment conducted by teams and diagnosis is often required for children and young people to access supports and services through neurodevelopmental teams. National specialist services specialise in conducting assessments in cases that are deemed diagnostically complicated or where specialist intervention is required. As such, referrals to these services have typically included a previous diagnostic assessment in a local service. Participants were recruited using convenience and snowball sampling. In order to be eligible, participants were required to have at least 3 years of post-qualification experience working with children and families and to be currently involved in clinical work. Interviews ranged between 43 and 69 min in duration, with a mean time of 59 min (standard deviation = 6.76). Twelve participants were female, and five were male. Years of experience ranged from 3 to >20 years. Ten participants were psychologists, two were paediatricians, two were psychiatrists and three were allied health professionals (e.g. nurses, speech and language therapists). We did not collect data on the number of clinical hours, which we regard as a limitation. Establishing the urban–rural divide proved challenging because national services saw young people from all over the United Kingdom.

### Data analysis

Data were analysed using the thematic approach outlined in Braun and Clarke (2006). BC and RD held regular meetings to discuss the data. BC has experience working in a neurodevelopmental team, and RD is an expert in child mental health and qualitative research. BC read each of the transcripts twice and listened to audio recordings to become familiarised with the data prior to coding. Transcripts were coded and recoded using NVivo 12. Codes and corresponding quotations were organised in NVivo 12 and also copied into a Microsoft Word Document. Codes were grouped into descriptive and analytical themes or patterns. These themes were reviewed and discussed in regular meetings between BC and RD. As the analysis indicates, we identified a wide array of perspectives on some topics.

### Community involvement statement

We received feedback on a draft of the paper from a parent who had experience with the autism referral pathway. Adjustments to terminology and technical language were made following this feedback.

## Results

We identified the following two overarching themes: (1) approaches to diagnosis and (2) elements of diagnosis.

### Approaches to diagnosis

#### Nature of diagnosis

A variety of attitudes were expressed about the utility of diagnosis. Most HCPs implicitly or explicitly suggested that diagnosis was an important tool in terms of treatment planning and opening access to supports. That is, some participants offered reflections on cases where a diagnosis was helpful in terms of giving the child and family access to groups, educational supports or, in the case of ADHD, medication. In addition, there were times when diagnosis seemed to resonate with patients at the level of identity. Nevertheless, some participants described hesitations around diagnosis. Two HCPs lamented the dominance of medical discourse in terms of describing the functional needs of children:

I think there’s value in trying to identify needs based on both diagnoses. I think it’s a shame that we’re in a medical model. The medical model has come into so many spheres that a child’s needs can’t be thought of as their needs. (Participant 08)

Issues of ‘construction’ also came into view:

We don’t often see this, but there are some occasions it’s partly colleagues some psychologists are not keen on the diagnostic sort of construction of a thing like ADHD. But there are sometimes literally children who are climbing up the walls in the clinics. [You] have them who actually can’t physically sit still. (Participant 14)

This speaks to a common tension faced by HCPs and researchers alike, which is between the acknowledgement that the ‘object’ (i.e. diagnosis) can be both an expression of underlying biological influences and also shaped by social processes.

Overall, participants tended to suggest that a diagnostic ‘label’ carried considerable weight and, as such, required a robust assessment. This involved standardised assessments, reports from multiple sources, detailing of the family history within the context of a multidisciplinary team. Overall, there was a sense in which once a diagnostic label is applied; it was difficult to remove:

Our motto is to not label if we’re not sure. And get another look, another opinion because once you label, it is difficult to un-label them. (Participant 01)

#### Application of diagnosis: natural and pragmatic

Most HCPs tended to speak of autism and ADHD as natural ways of describing some children’s presenting differences. Phrases such as ‘fairly straightforward’ or ‘barn door’ were sometimes applied to cases where the child had ‘classic’ features of either condition:

. . . in the old Rutter sort of phrase, you know the receptionist could diagnose the child because they’ve got every single characteristic that you classically sort of read about. (Participant 14)

Conversely, there were cases where the ill-fit of these categories was apparent. For example, when reflecting on a recent case, one HCP explained,

. . . there was just nothing that . . . [laughter] . . . There’s nothing that said me ADHD. So he’s not a restless, fidgety boy, he does some stimulating behaviours, but they’re quite specific, and he’s not on the go all the time, and he’s not moving his concentration and attention seems appropriate to his cognitive levels. (Participant 03)

Yet establishing whether a child fulfilled diagnostic thresholds was often not straightforward. Diagnostic decisions seemed to be complicated by a range of factors, including developmental history, genetic or mental health comorbidities, or ‘subtle’ features. When responding to these cases, diagnostic decisions tended to hinge more explicitly on pragmatic considerations such as whether the child or the family would benefit from the diagnosis.

Natural and uncertain cases bring to the forefront competing desires or requirements on the ‘object’ (i.e. autism or ADHD). On one hand, there is a desire to apply diagnosis consistently. Among other things (e.g. treatment planning), this desire safeguards services from saturation and protects the integrity of the classification. However, there is also a requirement for the diagnosis to be useful. When the child’s behaviour is a fit, the diagnosis is ‘natural’, and these desires can proceed with little interruption. However, in uncertain cases, there was sometimes a need to relax diagnostic thresholds to ensure the child could have access to services. This is, of course, compounded by organisational conventions which require a neurodevelopmental diagnosis to gain access to support.

Participants were almost unanimous in the view that the availability of supports and services would not influence diagnostic decisions in terms of withholding a diagnosis or not ‘adequately describing the child’s needs’.

Another topic on which participants seemed unanimous on was that symptoms, behaviours and characteristics needed to have an impact on functioning. As we will see in the next section, this criterion was used by at least one participant to justify removing a diagnosis at the request of the young person.

#### Revisiting conceptualisations: sub-optimal practice and unhelpful diagnoses

Despite perceptions that diagnoses were challenging to change, most participants indicated that they had been involved in revisiting or revising a diagnosis of autism or ADHD. Two reasons for revisiting a child’s diagnosis were identified as follows: (1) sub-optimal practice and (2) the diagnosis was considered unhelpful. Regarding the former, these assessments were often conducted by private practitioners or inpatient units and sub-optimal practices ranged from the practitioner not meeting the child to diagnosis without standardised assessments:

They [private consultant] was diagnosing autism sometimes not even seeing the child just talking to the parents. And nothing more than sixty minutes maximum, and we had [hundreds]of kids on their books, with diagnoses varying from social communication differences to attachment to autism spectrum, Asperger’s, pathological demand avoidance; there was no rhyme or reason why or how these came around . . . [. . .] . . . there was no standard [assessment]. None of those school-age kids had ADOS^1^ or ADI; it was just a clinician meeting them and the family and getting a school report and making the diagnosis. (Participant 01)

This challenge seemed more acute for clinicians in local services. However, it is important to note, as the quotation above indicates, that most of these participants indicated that they would not dismiss a private diagnosis out of hand. Second, as discussed above, throughout the data, participants suggested that diagnosis should be helpful to the individual. Therefore, part of the discussion regarding revisiting a diagnosis focused on cases where the individual felt that it was no longer useful. HCPs indicated that people want diagnoses ‘changed’ or ‘removed’ for a myriad of reasons. Sometimes, for instance, the diagnosis no longer resonated with the child or family. Else, there were concerns regarding prejudice or exclusion from certain professions (e.g. Military). In one case, the diagnostic criterion regarding functional impairment was occasionally operationalised to remove a diagnosis.

#### Sorting by autism and ADHD

There was agreement among HCPs that a child could meet diagnostic thresholds for both autism and ADHD concurrently. Thus, sometimes diagnostic teams give a dual diagnosis or recommend further assessment for either condition. Again, diagnostic decisions were shaped by both a degree of pragmatism and the extent to which the child’s behaviour seemed a ‘more’ natural fit with either diagnosis:

We certainly do diagnose both. The problem when a child presents, and it looks like they’ve got both is you sort of have to decide which one to do first practically, although they can sequentially run. And sometimes you’re sort of, so this is autism with some ADHD, or this is ADHD he’s a bit on the autistic spectrum. It sort of depends on what’s presenting with the most difficulty. (Participant 04)

There was a sense that social problems, inattention, hyperactivity or sensory seeking behaviours could stem from either autism or ADHD. We identified three considerations, which feed into differential conceptualisation: (1) form/nature of behaviours, (2) function of behaviours and (3) tacit factors. Regarding the form and nature of behaviours, although reference to social problems was not uncommon in children with autism and ADHD. However, social problems caused by interrupting peers, as opposed to a lack of overt interest, tended to be viewed as an expression of ADHD rather than autism.

By contrast, social problems resulting from sensory sensitivities were considered by one HCP as a subtle potential marker for autism. Other transdiagnostic developmental differences such as inattention and hyperactivity tended to be more challenging to untangle in terms of form:

It’s harder because it could be lots of reasons why someone’s inattentive or hyperactive. (Participant 11)

### Elements of assessments

#### Contextualising standardised assessments

Somewhat predictably, all participants indicated that standardised assessment tools were important sources of information in the diagnostic workup. There were some variations between services regarding the nature of these tools. This seemed driven by resourcing factors and clinician preference. Nevertheless, frequently cited measures included ADOS (Lord et al., 2012), ADI-R (Lord et al., 1994), Developmental Diagnostic Dimensional Interview–Short Version (Skuse et al., 2004), Social Communication Questionnaire (SCQ; Rutter et al., 2003) for autism, and parent and teacher versions of the DAWBA, SDQ (Goodman, Ford, Richards, et al., 2000), Connor’s rating scale (Conners, 2008) and QbTest (QbTech Ltd, Stockholm, Sweden) for ADHD. For one clinician, there was a sense in which these tools offered external validation of clinical opinion:

it [ADOS] enhances what you already think. So usually, if you think, ‘oh, this child feels like they’ve got autism’, you do an ADOS, and it clearly shows that they have. (Participant 10)

Participants seemed unanimous in the view that these assessments should be considered with other sources of information:

It’s about the ADOS, the ADI and other observations and assessments we need to do. We wouldn’t ever just take an ADOS and say yes, they meet criteria because there could be other reasons why that is. Likewise, if somebody does an ADOS and they score at below cut-off, we wouldn’t say they definitely haven’t got autism because we might take a developmental history and see them in other settings where they do look to have autism. (Participant 02)

Indeed, an array of ‘other reasons’ for a child’s performance on these measures can be found throughout the data. In terms of child and family factors, some HCPs indicated that these tools become more challenging to interpret when there is a comorbid learning disability or mental health problem (e.g. depression). The core difficulty here seemed to be that other conditions could explain certain symptoms:

[the child] was significantly depressed, and they did an ADOS while they were significantly depressed. And of course, eye contact was poor. Of course, they had the flat affect and all of that. (Participant 02)

Ultimately, there was a sense that scores derived from these assessments should be used with a degree of caution:

instruments are good at eliciting information; scores are very dangerous . . . [. . .] . . . it’s also just true that, in a sense of understanding data, scores on instruments, for example, diagnostic instruments like the ADI and the ADOS, the SCQ, and whatever instrument it is measuring whatever; They will help you least where you need the help most. (Participant 14)

Various other structured assessments for adjacent abilities or experiences can be found throughout the data. These include projective assessments, questionnaires about anxiety or uncertainty and cognitive assessments.

#### Triangulating material

Most HCPs accentuated the importance of collecting and triangulating information from multiple sources and stakeholders to get a sense of the child’s behaviour in different contexts. Other sources included professionals in adjacent mental health or social work teams, medical professionals, the child’s extended family (e.g. grandparents) and schools. Diagnostic challenges ensued when practitioners were unable to obtain a coherent developmental history. In these situations, school observations were described as an important source of information.

Indeed, most participants emphasised the importance of school reports and school observations. Yet, there were some critical comments about these reports. As one interviewee put it:

schools can sometimes present things as more positive than they are or have decided in their heads actually this is all parenting . . . [. . .] . . . And so when we’re asking for information, you know, ‘how they are at school’, ‘oh we don’t have any problems this is all parenting this is all Mum bringing him in’. (Participant 02)

#### Organisational factors

There was some variation in assessment practices between national specialist services with some having additional supports such as classroom-based in the service and access different standardised instruments. Nevertheless, the critical difference between national specialist and local services seemed to be time and remit and resourcing to conduct certain assessments:

We have the time and the more in-depth tools to really look into kids who may have more subtle presentations or where there’s a question about comorbidities or alternative explanations. (Participant 09)

Several participants emphasised the importance of cognitive assessments. Yet, the arrangements and resourcing issues meant that, in some settings, cognitive assessments were typically conducted by educational psychologists in schools. There were some critical comments about the support the team were receiving in this regard:

If the ed psych wasn’t up for doing a formal cognitive test which is also fairly often the case. Mostly, in fact. Then we could do that ourselves. We don’t . . . It’s not a major part of our remit really diagnosing LD, but if we’re already assessing for something else and it’s really critical for us, we would do that. (Participant 17)

As discussed elsewhere, the availability of supports did not seem to factor into decision-making in terms of withholding a diagnosis. However, for some national specialist HCPs, resourcing issues in local services meant that recommendations occasionally had to be negotiated:

We are a specialist second opinion team. I think what does slightly influence us, and we try not to, but it requires a bit more negotiation is our recommendations. So if you’ve got someone with co-occurrent anxiety a co-morbid anxiety disorder, and you wanted to arrange some cognitive behavioural therapy, then I guess whether that’s possible in local services needs a bit of negotiating. So yeah you have to think about what you’re recommending. (Participant 11)

HCPs in local services described referring children to national specialist services when they were ‘stuck’, or there was a ‘subtle’ presentation, complicated by comorbidities or developmental history. Yet, some tensions were identified between the levels of care when there were diagnostic disagreements. Reflecting on this, one HCP in a local service stated,

Sometimes what happens is when you go to tertiary centres they don’t have the system knowledge, and parents don’t tell them whereas here when I open the electronic patient record I know what happened with the health visiting, school nursing, general practitioner, safeguarding team. When you go off somewhere else, you don’t have that. And we also got informal networks and arrangements where I can pick up the phone and talk to people without revealing names and things. (Participant 01)

National specialist HCPs did not, in general, tend to speak of strategies to alleviate these tensions. Although one HCP did indicate that informal conversations with local teams can help bring the services together:

We try as much as possible to work with the local teams, so if I’m going to be doing a speech and language assessment, I will always contact the department . . . [. . .] . . . I feel sometimes just having a ten-minute chat with them just breaks down the barrier. (Participant 10)

## Discussion

This study sought to provide information about clinical perspectives on assessment practices for autism and ADHD in a sample of experienced HCPs in the United Kingdom. We identified two overarching themes (1) *approach to diagnosis* and (2) *elements of assessment*. An array of attitudes was expressed regarding the nature of diagnosis, with some critical comments regarding the preponderance of medical discourses as the mode of describing the functional needs of children. Nevertheless, diagnostic decisions tended to centre on (a) whether a child’s behaviour was a natural fit with diagnostic criteria or (b) whether a diagnosis would benefit the child or family. Indeed, a pragmatic approach to diagnosis was a persistent thread throughout the interviews when discussing instances of revisiting a diagnosis or differentiating between autism and ADHD. Importantly, however, a pragmatic approach tended to be adopted in diagnostically uncertain cases, and all participants accentuated the importance of a robust assessment. Relatedly, all participants indicated that they used standardised assessments in the diagnostic workup. However, participants emphasised that these tools need to be considered in conjunction with other forms of information due to psychometric, clinician or child and family factors. Somewhat predictably, inconsistencies between stakeholders or discrepancies between the various forms of information were not uncommon and framed as problematic. Assessments of adjacent abilities or experiences seemed to figure in the diagnostic workup, although there was some variation regarding the nature of these assessments across the various teams and services. Nonetheless, cognitive assessments were discussed relatively often. In local teams, resourcing issues came into view regarding support for conducting cognitive assessments.

### Situating the findings within the literature

Hacking (1999) suggests that there is room for ‘constructionist’ and ‘biological’ considerations in discussions regarding the nature of diagnosis. In general, critical comments about medical discourses did not seem to signal scepticism about the presumed biomedical aetiology of these conditions. Instead, these criticisms spotlighted organisational arrangements which implicitly and explicitly favour neurodevelopmental diagnosis over a functional description of the child’s needs in terms of allocating resources. Furthermore, these critiques also seemed to clarify the level of diagnostic clarity that can be attained through current assessment practices.

Elsewhere (e.g. Rogers et al., 2016; Skellern et al., 2005), it has been reported that HCPs occasionally relax diagnostic thresholds or ‘upgrade’ to a neurodevelopmental diagnosis in diagnostically ambiguous cases. This study offers some support to these findings in that there were occasions where diagnostic decisions seem to be made on pragmatic grounds rather than strict adherence to diagnostic thresholds. This, Rogers et al. (2016) suggested, is problematic because it might indicate that diagnostic classifications are not being applied in a consistent manner. We propose that the process of ‘upgrading’ speaks to competing desires for the object (i.e. autism or ADHD), which might be uneven across research and clinical discourses. Both discourses value consistency and practical utility. Unsurprisingly, these requirements are fulfilled in tandem when the application or ill-fit of a diagnosis is ‘natural’ (i.e. presence of ostensive features or lack thereof that tally with nosological criteria).

Yet, it is a clinical reality that some ‘pragmatic’ adjustments might be required in diagnostically complicated cases to help ensure the child’s needs are met. Therefore, from a theoretical standpoint, it seems likely that over time, pragmatic applications of diagnosis will shape what is considered a ‘natural fit’. Indeed, the history of both autism and ADHD indicate that this is the case. Importantly, however, pragmatic adjustments are not confined to clinical discourses. A desire for statistical power, for instance, means that adjustments are routinely made in research contexts. For example, screening tools as often used as a proxy for diagnosis, and there is substantial methodological variation between epidemiological studies about what qualifies as a positive incidence of a diagnosis. These research practices, among others, also shape our ideas about diagnosis. Nevertheless, broadly speaking, we agree with others (e.g. Skellern et al., 2005) who have suggested that structuring services to meet individual needs, rather than specific diagnoses, is a laudable goal. Using symptom mapping approaches (Borsboom & Cramer, 2013; Coughlan et al., 2021) and applying these to service configuration might be a fruitful approach. However, there is enthusiasm for more dimensional approaches to neurodevelopment with programmes such as Research Domain Criteria looking promising (Clark et al., 2017). However, the feasibility of such an approach applied to service organisation remains an open question.

Maynard and Turowetz (i.e. Maynard & Turowetz, 2017, 2019; Turowetz & Maynard, 2016, 2019) trace a number of narrative practices involved in evidencing a diagnosis of autism. Although we did not apply conversation analysis to this study, examples of practices such as ‘instantiations’ (e.g. ‘[child] was much more fidgety and fiddly and would kind of dart off on subjects but not in the way that you might find a youngster with autism’) and tendencies (i.e. ‘can’t keep focussed on the lesson so [they] get into trouble because [they] don’t know what is being expected’) can be found throughout the data. This lends some support for these aspects of the conceptual model outlined by Maynard and Turowetz (2017) regarding the structure of autism diagnosis. Also, it offers tentative support that some elements might extent to similar conditions such as ADHD. As discussed in the literature review, Turowetz and Maynard suggest that, in psychological reports, diagnosis is described as a fundamental property of the child and assessment factors (e.g. the clinician and assessment items) receive little attention or are presented as static. Yet, psychological reports and feedback meetings are two discrete practices, wherein the HCP is tasked with evidencing diagnostic decisions. In this study, the variability of assessment factors was underscored by several HCPs when reflecting on routine clinical work. Therefore, it seems that assessment factors are not considered to be static or irrelevant. Still, this study is not epistemologically positioned to offer firm evidence on this point. Further work using a combination of ethnographic and interview methods with professionals and patients might provide important insights on this issue.

Several of the HCPs offered reflections on cases where they had revisited a diagnosis. Psychometric studies (e.g. Algorta et al., 2016; Chang et al., 2016; Hall et al., 2019; Lord et al., 2006; Ozonoff et al., 2015; Randall et al., 2018; Woolfenden et al., 2012) overwhelmingly indicate that diagnostic decisions should not be based solely on the outcomes of standardised assessments. However, the fact that some local clinicians reported encountering cases referred to their services where some children had received a diagnosis without any standardised assessments and after a short period is potentially concerning. Putting aside the desire for consistency, which seems inevitably negotiated by such an approach to diagnosis, it is also unclear whether this application of diagnosis would be helpful. For example, professional and institutional criteria might mean that local teams or services might not accept a diagnosis, where standardised assessment reports are not available. As such, the child might be no better off in terms of access to supports for the diagnosis. Still, best practice guidance material (i.e. The National Institute for Health and Care Excellence, 2011, 2018) in the United Kingdom does not actually require that autism or ADHD-specific tools are used in the diagnostic workup. Therefore, further work which explores how the idea of a robust or adequate assessment is developed and negotiated between professionals might be beneficial.

As discussed, HCPs in survey studies tend to indicate neutral response to questions regarding the superiority of clinical judgement over standardised assessment or vice versa (Danielson et al., 2019; Jensen-Doss & Hawley, 2010). In this study, without exception, HCPs indicated that scores from standardised assessments needed to be considered with other forms of information and clinical judgement. For the HCPs in this study, standardised assessments are a key component of clinical judgement, but ultimately clinical judgement is required to consider the outcomes of these assessments. Survey work indicates that conflicts between clinical judgement and standardised assessments are not uncommon (Crane et al., 2019). The results indicate that an array of factors shape how HCPs consider the outcomes of these assessments, including clinician factors and comorbid difficulties.

Several studies (De Los Reyes, 2013; De Los Reyes et al., 2013, 2015; De Los Reyes & Kazdin, 2005) have shown conflicts between stakeholder reports and inconsistencies are relatively common. In line with these findings, discrepancies were discussed relatively often in this study and often created obstacles to diagnosis. Elsewhere, De Los Reyes (2013) reviewed several explanatory models of informant discrepancies. These explanations include informant biases, measurement error and variations in perspectives on the behaviour due to asymmetrical contextual factors (e.g. school vs home). Yet, one of the most clinically important findings from this study is that several explanations were used to understand these discrepancies. Therefore, there was not one clear heuristic for understanding uneven reports.

Finally, the finding that some HCPs were not getting adequate support for cognitive assessments is concerning. This is within a context where 42% of parents of children with autism indicate that their child was refused an assessment of special educational needs when requested (All Party Parliamentary Group on Autism, 2017). In the United Kingdom, educational psychologists tend to be employed by local authorities. A recent report commissioned by the Department for Education (2019) identified significant shortages and difficulties recruiting educational psychologists. In response, the Department of Education has pledged £31.6 million to fund 600 training places in educational psychology. How this increase in funding will be felt by schools and those awaiting assessments remains to be seen.

### Strengths and limitations

One of the strengths of this study is that participants were recruited from a range of services, including national neurodevelopmental services. Furthermore, the flexible topic enabled us to capture a wide range of perspectives on novel and unanticipated topics (e.g. parental knowledge of assessment tools). Still, the fact that eight participants worked in national specialist services meant that some potential topics (e.g. resourcing issues) were not as prominent in the data. Further only one parent provided feedback on the paper, which we regard as a material limitation. It is also worth noting that our research focused on autism and ADHD, and therefore, it is not clear whether the findings apply to other neurodevelopmental conditions. Finally, our sampling techniques (i.e. convenience and snowball sampling) and the relatively high number of psychologists (n = 10) might mean that the perspectives discussed in this study might not represent other professions.

## Conclusion

This study has identified some of the contextual factors that shape assessment practices for autism and ADHD and, as such, adds to the literature on neurodevelopmental assessment. One of the main findings is that diagnosis is typically applied in two ways, which we call natural and pragmatic. Although often problematised, pragmatic adjustments are common in both clinical and research. However, we agree that structuring services based on individual need, rather than diagnosis, is a potentially sensible goal. Moreover, it would seem crucial that decisions about service configuration are made in conjunction with experts-by-experience to best understand how to meet their need and wishes. In addition, our results indicate that standardised assessments are important in neurodevelopmental assessment. However, an array of factors influences the utility of these tests, and different approaches are required to negotiate tensions between discrepant forms of information.

## Supplemental Material

sj-docx-1-aut-10.1177_13623613221121413 – Supplemental material for ‘Instruments are good at eliciting information; scores are very dangerous’: The perspectives of clinical professionals regarding neurodevelopmental assessment Supplemental material, sj-docx-1-aut-10.1177_13623613221121413 for ‘Instruments are good at eliciting information; scores are very dangerous’: The perspectives of clinical professionals regarding neurodevelopmental assessment by Barry Coughlan, Matt Woolgar, Emma JL Weisblatt and Robbie Duschinsky in Autism

## Notes

1. Throughout the interviews, clinicians tended to use ADOS and ADI as shorthand for the aforementioned ADOS-2 and ADI-R.
