# Supplementary material for: ‘Instruments are good at eliciting information; scores are very dangerous’: The perspectives of clinical professionals regarding neurodevelopmental assessment
Source: Autism. 2022 Sep 25;27(4):905–15. doi: 10.1177/13623613221121413 (PMC10115934; doi:10.1177/13623613221121413)
Supplement: sj-docx-1-aut-10.1177_13623613221121413 – Supplemental material for ‘Instruments are good at eliciting information; scores are very dangerous’: The perspectives of clinical professionals regarding neurodevelopmental assessment [file sj-docx-1-aut-10.1177_13623613221121413.docx]

#### Supplement 1. Full study interview guide

Introduction:

I would like to ask you some questions about your training and background, your routine clinical activities, and your experiences and views on the diagnostic process for developmental conditions in children. I would like to remind you not to disclose any personally identifiable information about any individuals, child or families you work with during the interview.

Background

Can you give me a brief overview of your current position?

***Probe*** for experience related to child development

How long have you been working as a (GP, psychologist, psychiatrist etc)?

***Probe*** for length of time working with children and families

Where and when did you do your clinical training?

***Probe*** for General Practitioner (GP) training vs medical training

Clinical work

Without revealing any personally identifiable information, can you tell me about a case where you conceptualised the child’s features as relating to autism?

**Probe** action steps

**Probe** specific difficulties with working with this child

**Probe** for informal assessment (e.g. familiarity with things in the past)

**Probe** assessments (I know X is saturated with screening tools/assessments; observations)

**Probe** for level of clarity (how certain or uncertain were you about X)

**Repeat for ADHD and attachment related**

If not clear, can you give me an overview of your involvement in relation to identification and assessment of developmental (or behavioural) difficulties?

**Probe** for assessment tools, observations and focus on developmental histories, team-based approach etc.

**If GP, probe** for screening tools.

Can you think of a specific instance where there was uncertainty regarding the nature of a child’s difficulties/diagnosis?

**Probe** for description

**Probe** for action steps

Are there other factors that contributed/routinely contribute to the decision you made (e.g. system factors, availability of supports and services)?

Have you ever been involved with overturning or changing a child’s diagnosis?

Case Study Questions

**Questions for participants with Case Study 1,**

What are your initial thoughts on the case?

What questions would you ask A) Robert B) Parents (Tim & Linda)

What would you advise as the next steps?

If the participant deems onward referral is necessary

what information would you put in the referral letter?

Why did X resonate with you?

How easy have you found it to get external help

What did you find yourself drawing on to make sense of Roberts presenting difficulties? (i.e. other similar cases, diagnostic criteria, theory etc)

If the child had a private diagnosis of autism, would that influence how you think about the case?

If you were unsure about the nature of the child’s difficulties, are there other professionals you would consult with?

**Questions for participants with Case study 2.**

What are your initial thoughts on the case?

**Probe** for possible diagnosis, working clinical hypotheses, formulation

If the participant states a specific diagnosis/conceptualisation

What features of the case helped you to arrive at that conclusion?

Are there other conditions you considered?

**Probe**, if so what?

**Probe** how the participant differentiated

If the participant indicates that it could be multiple conditions

You mentioned that it could be X or Y, how would you differentiate?

What assessments/sources of information would be useful in helping you to reach your decision?

If the participant indicates there isn’t enough information to make the decision

What information would help you make a decision regarding the nature of the child’s difficulties?

Are there specific assessments/ theories you would use?

In your view, is there need for further assessment?

If Yes, what are the next steps in terms of assessment?

**Probe** for risk assessment

**Probe** cognitive assessment

**Probe** sources of information

Are there any frameworks/ theories you used to understand the nature of the child’s presenting difficulties?

**Probe** for specific models/ theories

**Probe** for influential book/text/ or talks

If the child had a private diagnosis of autism, would that influence how you think about the case?

If you were unsure about the nature of the child’s difficulties, are there other professionals you would consult with?

Referral Pathways Information

In your experience, what do you consider the early markers of autism?

*Probe for symptoms, features, predisposing events, comorbid conditions, family history*

**repeat for ADHD, Attachment related difficulties.

Rotate order each interview

Questions for Primary Care Clinicians

Have you ever referred a child to a CAMHS or relevant assessment service as you suspected the child may a developmental condition or behavioural difficulty?

If yes, what information did you include in your referral letter?

Have you ever had a referral of this nature not accepted in the first instance?

If yes, why was the referral not accepted?

On a scale of 1-10, how easy have you found it to get formal assessment for a child you think refer a child who you think may have autism?

**Repeat** for attachment problems

**Repeat** for ADHD

Have you experienced any challenges to referring a child who you think may have autism?

**Repeat** for attachment problems

**Repeat** for ADHD

What, if anything could be done to make the referral pathway, clearer?

On a scale of 1-10, how confident do you feel that you will be able to get the appropriate a) assessment b) support for a child with autism, adhd, attachment problems.

Questions for non-primary care clinicians

Do you receive many referrals to your service from GPs?

If yes, how would you describe the quality of these referrals?

Probe what’s in a good/less good referral

If no, where do you get the majority of referrals from

How would you describe the quality of these referrals?

What information would constitute a sufficient referral (i.e. allows you to make your decision regarding acceptance for subsequent assessment/support)

What information could be collected by a GP to assist with the decision to accept or reject a referral to your service?

How do you think children and families experience the journey from primary care to your service?

Thinking about conditions with overlapping features, do you see value in differentiating autism from attachment-related conditions?

Do you see a meaningful distinction between the attachment disorders (RAD and Disinhibited) and attachment-related difficulties?

Similarly, when considering a diagnosis of autism and adhd, what value do you see in diagnosing both or trying to differentiate?

Finally, what in you view is the value in differentiating ADHD from attachment-related conditions?

Case Study

Robert (aged 6) was referred to CAMHS by his general practitioner due to concerns regarding his behaviour and overall development. Robert has made little progress, academically, since starting school one year ago and is described as having few friends. In addition, he has been growing increasingly aggressive towards his mother, Linda (33), and recently threw her laptop computer at a wall. According to Linda, his behavioural issues are longstanding, and she is finding it difficult to manage. Previously there have been safeguarding concerns and social work has been involved with the family.

A developmental history questionnaire revealed no significant issues with pregnancy or birth. Under the strengths section of the questionnaire, “none” was written. Robert achieved his motor milestones; however, his language development was delayed. Previously he received speech and language therapy in the community. The questionnaire also revealed that Robert has an older half sibling, Chris (14) who has a diagnosis of ADHD. The referral was accepted on the basis of the paper screen.

In the first instance, Robert was booked in for an unstructured behavioural/play assessment and offered a place on a parenting programme. Prior to the assessment, the team received a number of phone calls from Robert’s father, Tim (32). In the first phone call Tim said he would not attend the parenting course as parenting isn’t the issue and that he found the invitation insulting.

Robert was on the waiting list for just over 3 months. Over this period, Tim phoned the team a number of times to express his dissatisfaction with the waiting list. At one-point Linda phoned crying saying Robert was “out of control”.

One month later Robert attended the assessment. From the outset, Robert appeared distressed (i.e. crying). Linda made numerous attempts to comfort Robert, but he moved away in response to each of her approaches. At one-point Robert kicked out at Linda. There are what look like two distinctive episodes of hand-flapping.

During the assessment Robert presents as active (e.g. jumping up and down, throwing ball in the air) and moved from one activity to another in quick succession. Robert’s eye contact was fleeting, and he did not demonstrate the full range of facial expressions. In terms of conversation, Robert spoke in complex sentences, although the subject matter was a little repetitive and mainly around his favourite toy (Shopkins).

After the assessment, Tim and Linda completed the autism diagnostic interview (ADIR). The assessment lasted 2hrs 30 mins. When asked about development prior to three years, Linda disclosed that Robert lived with his grandmother beginning when he was 18 months old to just after his third birthday, as Linda and Tim were separated during this period. During the separation, Linda was an inpatient at a local mental health facility. Robert’s score on the ADI-R was just below the threshold for autism.
